# Kalinix^®^, an innovative, intelligent, non-invasive, and self-adaptive medical device for effective OSA management

**DOI:** 10.1007/s11325-026-03687-y

**Published:** 2026-04-29

**Authors:** Juan Luis Rodríguez Hermosa, Myriam Calle Rubio, Ernesto Delgado Cidranes, José Luis Álvarez-Sala Walther, Víctor Javier Montero Blasco, María Ángela Velacoracho Uriel, Baldomero Fernández Rondán

**Affiliations:** 1 Clinical Hospital of San Carlos, Madrid, Spain; 2 Faculty of Medicine, Complutense University of Madrid, Madrid, Spain; 3 CIMEG Clinic, Madrid, Spain; 4 Pain Unit, Hospital La Milagrosa, Madrid, Spain; 5 Torytrans S.L, Madrid, Spain; 6 Novategia S.L, Tres Cantos, Spain

**Keywords:** Innovative OSA therapy, Kalinix^®^, Non-invasive treatment, Clinically effective, Portable device, Patient comfort

## Abstract

**Purpose:**

Obstructive sleep apnea (OSA) is a rising prevalent disease mediated, in part, by the excessive relaxation of pharyngeal muscles that increases the vulnerability for pharyngeal collapse. At the therapeutic level, the reduced patient’s compliance of continuous positive airway pressure (CPAP) linked with the limitations of alternative OSA treatments, emphasizes the need for novel non-invasive and effective treatment options.

**Methods:**

This real-world interventional clinical evaluation aims to explore the viability and short-term performance of Kalinix^®^ in a clinically relevant OSA population representative of the routine clinical practice. Kalinix^®^ urge as an innovative, intelligent, non-invasive, multi-sensor and self-adaptive medical device developed to overcome the limitations of the current OSA treatments. Their clinical efficacy, under real-world conditions, was explored in this unicentric and interventional study that included 20 patients with moderate to severe OSA.

**Results:**

The clinical efficacy of Kalinix^®^ was demonstrated by the statistically significant improvements in key OSA clinical parameters, with 55% of patients experiencing improvements > 50% in apnea-hypopnea index (AHI). Additionally, 100% and 42.9% of patients with moderate OSA (baseline AHI ≤ 29) or severe OSA (baseline AHI 30–49) were classified as Kalinix^®^ responders (residual AHI < 15). Moreover, based on investigator-defined clinical criteria (AHI+ oxygen desaturation index [ODI], AHI+ percentage of time with an oxygen saturation < 90% [CT90], and AHI + ODI+CT90), nearly 40% of patients were categorized as good treatment responders. In addition to the clinical benefits observed with Kalinix^®^, 100% of patients reported satisfaction with the device ergonomics, portability, and performance.

**Conclusion:**

Kalinix^®^ showed promising results in the treatment OSA and offers a non-invasive, comfortable, portable, effective, and safe therapeutic option that may contribute to achieving good long-term compliance and clinical outcomes.

**Supplementary Information:**

The online version contains supplementary material available at 10.1007/s11325-026-03687-y.

## Introduction

Obstructive sleep apnea (OSA) is an increasingly public health problem due to its high prevalence and health impact, especially in developed countries [1, 2]. Recent evidence estimates that nearly 46% of the population may experience some form of OSA, specifically as age increases [3].

OSA is characterized by recurrent episodes of partial or complete collapses of the pharyngeal airway during sleep, which results in apneas and hypopneas [4]. These transient events trigger intermittent hypoxia and hypercapnia, autonomic nervous system and metabolic abnormalities, as well as sleep fragmentation and non-restorative sleep [2–6]. OSA-associated dysfunction relates to increased health risks, particularly those affecting the cardiovascular and cerebrovascular systems [6].

At the pathophysiological level, although the precise mechanisms underlying OSA remain unclear, the increased anatomical predisposition to pharyngeal collapse (e.g. obesity or bony structures crowding the airway) and the excessive relaxation of pharyngeal muscles are key contributors [7–9]. OSA-patients experience a negative impact on their quality of life though excessive daytime sleepiness, decreased energy levels, workplace errors, impaired global cognition and mood changes [6, 10].

The most common treatment strategy for OSA includes sleep hygiene, weight loss, positional therapy and continuous positive airway pressure (CPAP) applied every night during sleep. However, factors like the device, the patient- device interface and the comfort are common challenges identified by patients, impacting short and long-term treatment adherence [6, 11]. Other common therapeutic options include oral appliances (e.g. mandibular advancement devices [MAD]) and surgical procedures [6]. Nevertheless, MAD devices are more commonly indicated to treat OSA in some subgroups of patients (e.g. symptomatic patients who refuse or do not tolerate CPAP) and represent the treatment of choice only in 18.1% of patients [12, 13], while OSA surgeries are frequently associated with high morbidity, long recovery times, and low patient acceptance [6]. In recent years, neurostimulation therapy has emerged as a promising alternative to CPAP, particularly with the development of non-invasive devices that provide higher comfort, ease of use and minimal side effects [11].

Due to reduced compliance with CPAP and the limitations of alternative OSA treatments, there is a growing need for novel non-invasive and effective therapeutic devices. Here, we explore the clinical efficacy of Kalinix^®^, an innovative, intelligent, non-invasive, multi-sensor and self-adaptive medical device developed to OSA management. Briefly, Kalinix^®^ collects sensory data (respiratory flow and air temperature) and uses an AI algorithm to predict apnea/hypopnea events. In response, Kalinix^®^ applies external electro-therapeutic stimuli (primarily targeting the genioglossus muscle) to promote upper airway patency and improve respiratory parameters in real time.

## Methods

### Study overview

The Kalinix^®^ project is a unicentric and interventional study designed to explore the therapeutic efficacy of a novel, intelligent, non-invasive, multi-sensor and self-adaptive medical device (Kalinix^®^) for the treatment of OSA, conducted from November 2021 to July 2023.

Kalinix^®^ was developed by Torytrans S.L., and the clinical study to evaluate device efficacy was performed at the Clinical Hospital of San Carlos (Madrid, Spain). The project follows the rules established in good clinical practice guidelines and was approved by the Ethics Committee of the Clinical Hospital of San Carlos (C.I. 20/485-EC_P) on July 24, 2021 and authorized by the Spanish Agency of Medicines and Medical Devices (AEMPS; 835/20/EC) on August 28, 2021. All participants signed an informed consent prior to the enrollment in the study, in accordance with the principles of the Declaration of Helsinki and Spain’s new Organic Law 3/2018 on the Protection of Personal Data and the Guarantee of Digital Rights, effective since December 7, 2018. To preserve patient confidentiality, each patient was identified using a unique code.

### Study design

The study was designed as a real-world interventional clinical evaluation with the aim of evaluating the viability and short-term clinical efficacy of Kalinix^®^ in a real-world setting comprising a clinically relevant OSA population representative of the routine clinical practice. To explore the real-world efficacy of Kalinix^®^ in OSA treatment, the sleep patterns/parameters of each patient were evaluated at home for a maximum of 7 days. In day 1, at medical consultation, a pre-treatment phase was performed for each patient to determine device tolerability and the optimal individual stimulus intensity settings for the device (intensity required for the initial and maximal upper airway opening) using 3D/4D sonography and elastography (MyLab™X90, Esaote’s ultrasound) studies, with and without Kalinix^®^ stimulation, during approximately 20 min. To ensure an individualized therapeutic effect and to evaluate tolerability, the optimal stimulus intensity settings were determined by identifying the range between the patient’s perception (intensity at which the patient first perceives the stimulation) and tolerance thresholds (highest intensity tolerated without discomfort), while evaluating simultaneously the real-time airway opening mediated by genioglossus muscle contraction upon Kalinix^**®**^ stimulation.

On day 2, the same procedure was performed to each patient to validate the persistence of therapeutic effects upon Kalinix^®^ exposure. On day 3 the baseline sleep without any therapeutic intervention was evaluated domiciliary using a monitoring device (polysomnography with two EEG channels [PSG; NOX T3]). Also, to guarantee that patients became familiar with the device during the trial, Kalinix^®^ was also used during the study without active treatment. From day 4 to a maximum of 7 days patients were equipped with monitoring devices and treated with Kalinix^®^ (treatment phase) to determine optimal parameters for each patient, in a procedure similar to CPAP titration, which reflects the maximum potential therapeutic benefit under optimal conditions.

### Sample size calculation

The minimum number of patients included in the study to guarantee sufficient statistical power to detect significant changes in the apnea-hypopnea index (AHI), the standard measure to classify OSA severity and to monitor therapeutic outcomes, was performed based on formal sample size calculation. The calculation considered the expected mean reduction in AHI of 16 events/hour (from a baseline of 30 to 14 events/hour after Kalinix^®^ therapy) and a maximum standard deviation of 20 events/hour, based on previous evidence, including studies closely aligned with the present intervention type (electrical stimulation for OSA) [14–16]. Additionally, using a significance level of 0.05 and a power of 80% in a paired design (same patients before and after treatment), the minimum required sample size was calculated with the following formula:

$$\:n=\frac{2\left({Z}_{\alpha\:}+{Z}_{\beta\:}{)}^{2}\cdot\:{S}^{2}\right.}{{d}^{2}}$$

n: number of subjects required in each group.

Zα: Z value for the desired significance level.

Zβ: Z value for the desired statistic power.

S2: Variance of the variable of control or reference group.

d: minimum difference between groups to detect.

### Patient selection

Patients with a recent diagnosis of moderate or severe OSA in the Sleep unit of Clinical Hospital of San Carlos were included in the study (*n* = 20). For this purpose, patient medical and previously conducted sleep studies performed as part of routine clinical practice were reviewed and no additional tests were performed to select patients to the study. Selected patients had previously declined CPAP therapy at the time of study inclusion.

The inclusion criteria defined in the study were: patients of both genders, aged > 18 years, with a confirmed OSA diagnosis by PSG presenting AHI values ranging from 15 to 29.9 per hour (moderate OSA) or AHI > 30 per hour (severe OSA). To avoid potential bias, the criteria for patient exclusion included: arrhythmia and atrioventricular blocks; pacemaker use; body mass index (BMI) > 35 kg/m^2^; neuromuscular diseases; craniofacial malformations or oropharyngeal tumors; treatment with muscle relaxants, sleep inducers, melatonin or barbiturates; patients suffering from insomnia, restless legs syndrome, severe mental disorders, hypothyroidism, allergies to latex, tape, or plastics, under oxygen therapy from more than 15 h per day, CPAP or nocturnal oxygen therapy, pregnancy or breastfeeding; end-stage cancer, severe liver disease or severe/end-stage renal failure (glomerular filtration rate < 30 mL/min).

After reviewing the medical history and confirming that all the inclusion criteria were met, patients were informed both orally and in writing, about the step by step, study design. Information regarding data protection regulations was also provided. Once the patient had confirmed understanding of the study procedures, signed and dated consent forms were collected for each patient. It was assigned to each patient a unique sequential identifier to preserve anonymity.

### Device characteristics

**Fig. 1:**
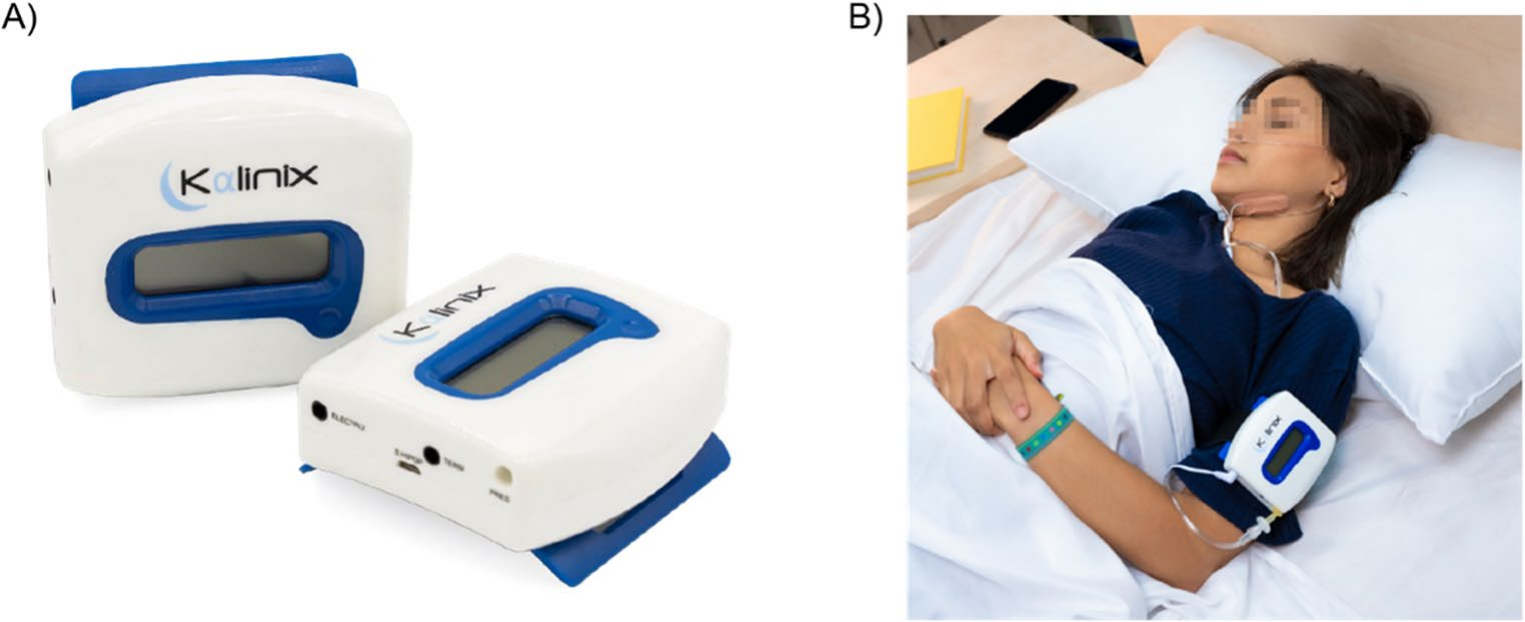
Kalinix^®^ device was developed as an innovative, non-invasive, multi-sensor, self-adaptive device and designed to predict respiratory events in real time and to apply a targeted electrotherapeutic stimulation to the lingual and perilingual (genioglossus) muscles to prevent the occurrence of apnea and hypopnea episodes during sleep. The phone-sized central device of Kalinix^®^ contains a processor responsible for predicting the apnea/hypopnea events and the electronics required to generate a real-time electrostimulation signal to prevent the events (**A**) and can be attached to the arm or chest using an easily adjustable strap. Kalinix^®^ connects to electrodes placed beneath the right and left jaw, which serve as electrostimulation applicators, and to a nasal cannula, which functions as a sensor for breathing patterns (**B**, illustrative image). The patient information collected by the sensors (respiratory rate and respiratory air temperature) were analyzed in real time by AI an algorithm to predict apnea/hypopnea episodes

Kalinix^®^ differs from existing neurostimulation approaches due to its innovative, intelligent, non-invasive, multi-sensor and self-adaptive design developed for the treatment of OSA (Fig. 1). It operates through real-time prediction of respiratory events and, each time an apnea/hypopnea episode is predicted during sleep, it continuously adapts the external electro-therapeutic stimulation to the lingual and perilingual muscles (genioglossus muscle) to avoid the episode.

Kalinix^®^ does not require surgical implantation and allows a dynamic, real-time adjustment of stimulation intensity and timing to favor the upper airway opening. This approach optimizes the upper airway muscle activation without causing perceptible discomfort to the patient and allows patients to move freely during sleep and avoid the inconveniences/discomfort of other OSA treatment systems. A detailed description of the device and the AI algorithm used for predict respiratory events can be found in the Supplementary Material.

### Data and clinical variables collected

Kalinix^®^ efficacy was assessed using primary clinical variables from polysomnography with two EEG channels (AHI, event duration, and event frequency), and secondary variables including oxygen desaturation index (ODI), percentage of time with an oxygen saturation < 90% (CT 90), oxygen saturation (SpO2%), BMI, neck and waist circumference, Mallampati score, respiratory rate, heart rate, patient satisfaction questionnaire, Epworth sleepiness scale (ESS) and evaluation of upper airway opening (mm, in the sitting and supine positions). Kalinix^®^-responders were defined as patients with a residual AHI < 15.

For comparative analyses between Kalinix^®^ and other therapeutic strategies, Kalinix^®^ efficacy versus CPAP/auto-adjusting positive airway pressure (APAP) was assessed based on baseline and post-treatment AHI values for both strategies. For non-CPAP comparisons, Kalinix^®^ efficacy was evaluated according to the success criteria defined in each selected study. Considering that many biological variables are assumed to follow a normal distribution, the eligibility of Kalinix^®^-treated patients for comparative analysis was defined according to rule of mean ± 2 standard deviations (SD) [17, 18]. Treatment efficacy within the selected patient subset was calculated as the percentage who met the predefined success criteria defined in each study, using the following formula:

$$Efficacy\;\left(\%\right)=\frac xy\times100$$

x: number of patients who met the success criterion.

y: number of patients in the subset.

### Statistical analysis

Data processing and analysis was performed using IBM SPSS Statistics version 26 statistical and R software version 4.4.3. The paired comparisons of quantitative parameters of two independent groups (pre- vs. post-Kalinix^®^ treatment) were conducted based on data distribution. Comparisons between variables that followed a normal distribution were performed using student’s t-test, and for asymmetric variables, the Wilcoxon test was applied. Additionally, to determine the statistical significance of patients who achieved improvements with Kalinix^®^ treatment, a null hypothesis test was employed. A p-value < 0.05 was considered statistically significant.

## Results

### Baseline patient demographics and characteristics

A total of 20 patients with moderate to severe OSA were included in the study. Kalinix^®^-treated patients had a mean age of 58.4 years (SD 14.0), being the majority male (70%). Additionally, 30.0% of the population were classified as having moderate OSA (AHI 15–29), 35.0% as severe OSA (AHI 30–49), and 30.0% as very severe OSA (AHI ≥ 50). All baseline patient demographics and clinical characteristics are compiled in Table 1.

**Table 1:** Summary of demographics and clinical characteristics of patients (*n* = 20) with a recent diagnosis of moderate or severe OSA that were included in the study.

Demographics and clinical characteristics	
Gender (male), n (%)	14 (70.0)
Age, mean (SD)	58.4 (14.0)
BMI, mean (SD)	27.7 (3.14)
BMI categories, n (%)	
(BMI 25–29.9.9)	14 (70.0)
Obesity (Class I-II [BMI 30–39.9.9])	3 (15.0)
Blood pressure, mean (SD)	
Systolic	127.3 (11.6)
Diastolic	77.2 (9.2)
Heart rate, mean (SD)	71.7 (4.9)
Respiratory rate, mean (SD)	13.4 (0.6)
Neck circumference, mean (SD)	41.8 (3.68)
Waist circumference, mean (SD)	98.8 (9.6)
Mallampati score, n (%)	
Class I: soft palate, uvula, and pillars are visible	0 (0)
Class II: soft palate and uvula are visible	3 (15.8)
Class III: only the soft palate and base of the uvula are visible	13 (68.4)
Class IV: only the hard palate is visible	3 (15.8)
ESS	
Mean (SD)	9.3 (5.6)
Score 0–6, n (%)	8 (40.0)
Score 7–8, n (%)	2 (10.0)
Score 9–24, n (%)	10 (50.0)
AHI (events/hour), n (%)	
AHI < 15	1 (5.0)
AHI 15–29	6 (30.0)
AHI 30–49	7 (35.0)
AHI ≥ 50	6 (30.0)
AHI, mean (SD)	23.8 (21.5)
CAI, mean (SD)	2.3 (3.7)
CT90 range, n (%)	
0	1 (5.0)
1–14	12 (60.0)
15–29	3 (15.0)
≥ 30	4 (20.0)
SpO_2_ %	
Mean (SD)	91.0 (3.1)
Minimum (SD)	78.1 (7.0)

*AHI* apnea-hypopnea index, *CAI* central apnea-hypopnea events per hour, *CH* central hypopnea, *CT90* percentage of time with an oxygen saturation < 90%, *ESS* Epworth sleepiness scale, *MA* mixed apnea, *OA* obstructive apnea, *OH* obstructive hypopnea, *SD* standard deviation, SpO2%, oxygen saturation

### Effects of Kalinix^®^ on upper airway dynamics

Before assessing Kalinix^®^ efficacy, the real-time evaluation of upper airway opening during stimulation was performed (Table 2). The Kalinix^®^-induced electrostimulation of the lingual and perilingual muscles (genioglossus muscle) promoted satisfactory improvements in upper airway opening, particulary in in the supine position (Table 2).

**Table 2:** Evaluation of the morphological and functional impact of Kalinix^®^ electrostimulation on upper airway opening in study patients, assessed in both sitting and supine positions

	Opening (mm), Mean (SD)	Improvement from baseline, Mean %
Sitting position		
Soft palate	3.1 (1.6)	53.9
Hard palate	3.5 (2.3)	38.0
**Supine position**		
Soft palate	3.9 (1.9)	70.1
Hard palate	2.5 (2.4)	49.9

*SD *standard deviation

### Clinical benefits of Kalinix^®^ on key OSA parameters

**Fig. 2:**
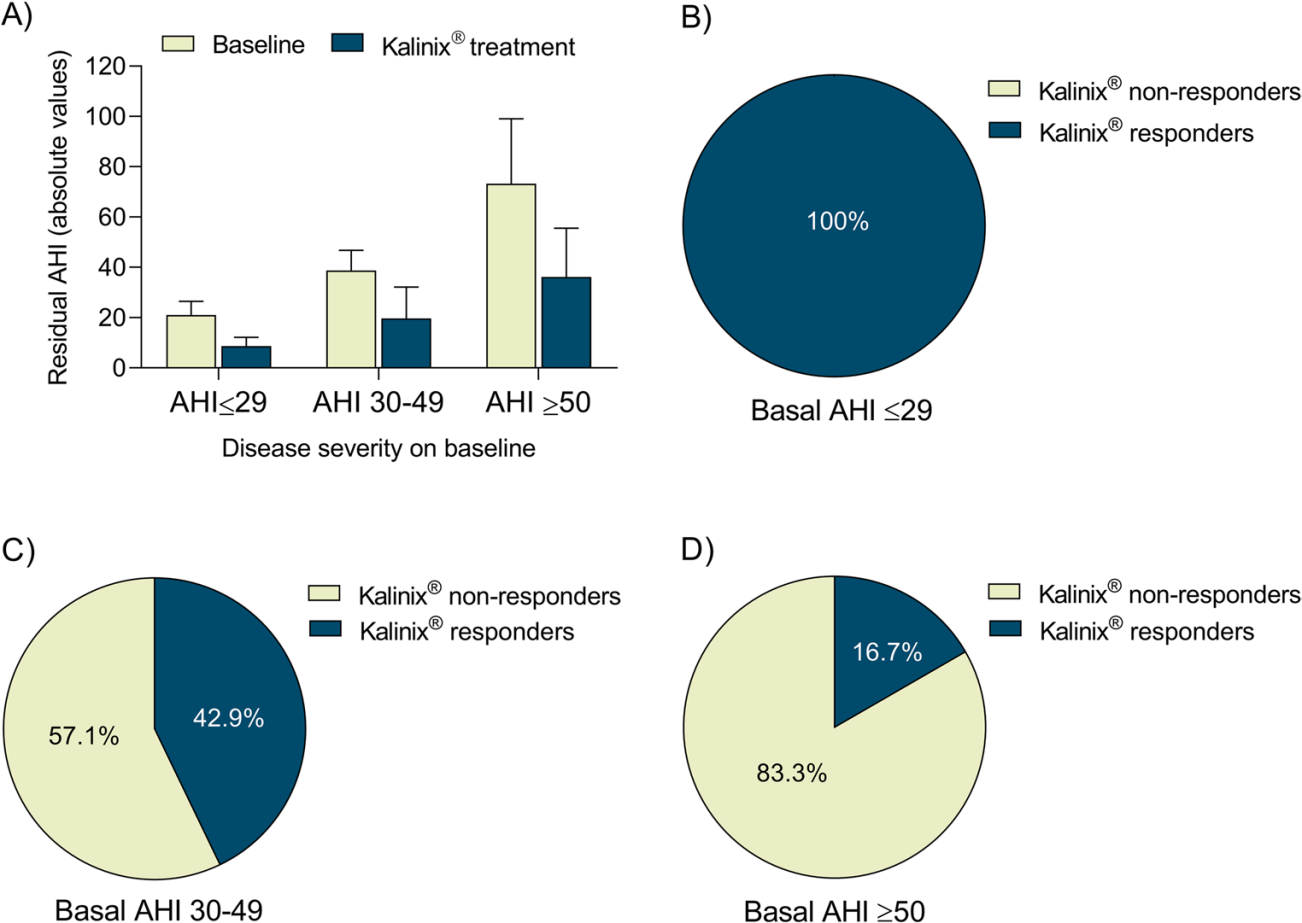
Improvements in AHI after Kalinix^®^ treatment in patients stratified according to disease severity at baseline (AHI ≤ 29, moderate OSA; AHI 30–49, severe OSA; AHI ≥ 50, very severe OSA). **A**) Residual AHI at baseline and upon Kalinix^®^ treatment, being the clinical response to Kalinix^®^ treatment positively correlated with disease severity; **B-D**) Percentage of non-responders (residual AHI > 15) and responders (residual AHI < 15) patients to Kalinix^®^ treatment by disease severity group. The percentage of responders was highest among patients with moderate OSA (100%), with a progressive decrease observed in patients with severe OSA (42.9%) and very severe OSA (16.7%). AHI apnea-hypopnea index, OSA obstructive sleep apnea

**Fig. 3:**
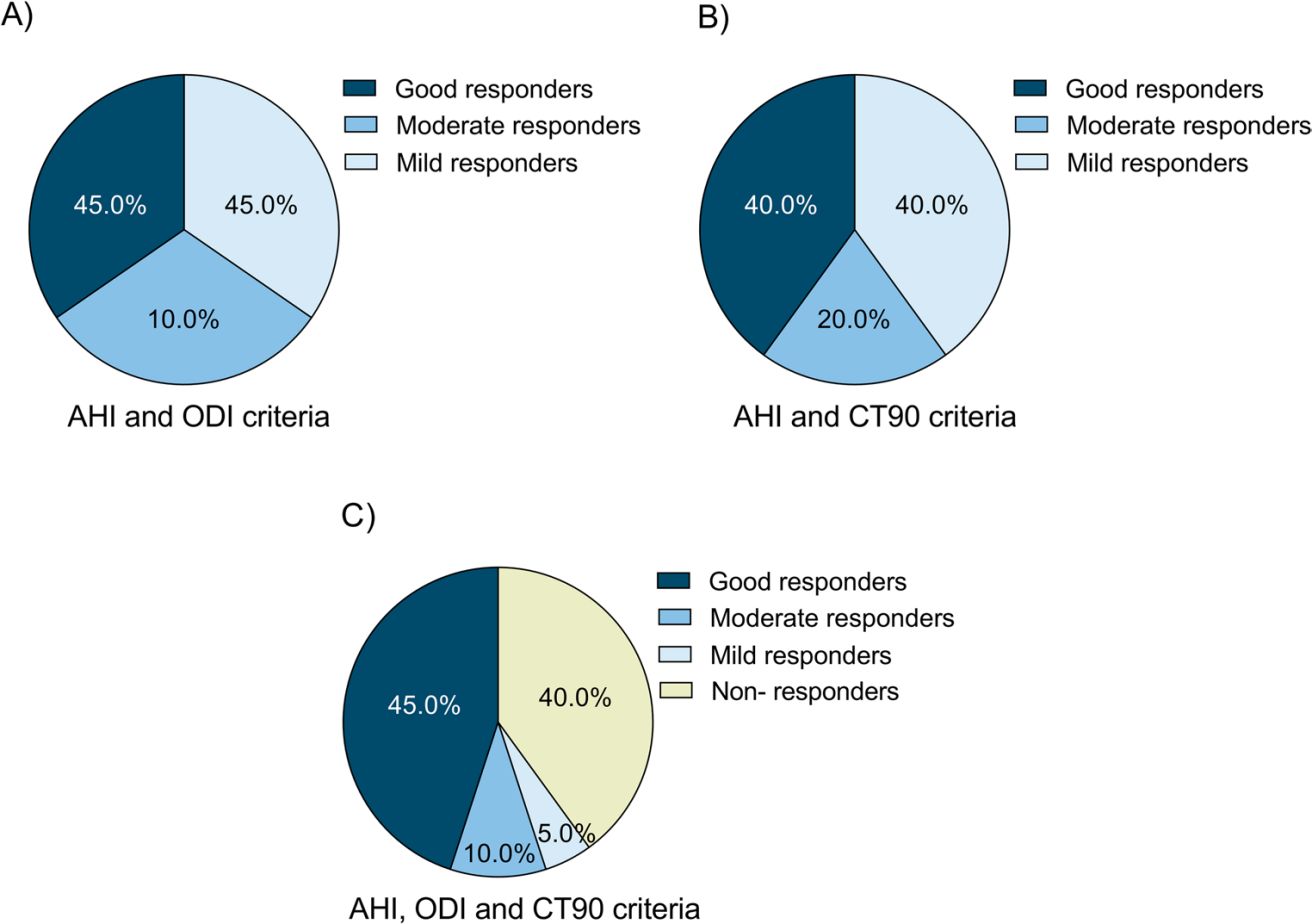
Distribution of OSA study population according to investigator-defined clinical criteria upon Kalinix^®^ treatment. Patients were classified based on changes in AHI, ODI, and CT90. (**A**) AHI and ODI criteria (good responders: AHI and ODI changes ≥ 50%, moderate responders: AHI or ODI changes ≥ 50%, mild responders: AHI and ODI changes < 50%); (**B**) AHI and CT90 criteria (good responders: AHI and CT90 changes ≥ 50%, moderate responders: AHI or CT90 changes ≥ 50%, mild responders: AHI and CT90 changes < 50%); (**C**) AHI, ODI and CT90 criteria (good responders: AHI changes ≥ 50% and change ≥ 50% in ODI and/or CT90, moderate responders: AHI change ≥ 50% and ODI and CT90 changes < 50%, mild responders: AHI change < 50% and ODI and/or CT90 changes ≥ 50%, non-responders: AHI, ODI and CT90 changes < 50%). Across the defined criteria, a substantial proportion of patients were classified as good responders following Kalinix^®^ treatment (45% according to AHI and ODI criteria, 40% according to AHI and CT90 criteria, and 45% according to combined AHI, ODI, and CT90 criteria). AHI apnea-hypopnea index, CT90 percentage of time with an oxygen saturation < 90%, ODI oxygen desaturation index, OSA obstructive sleep apnea

The clinical efficacy of Kalinix^®^, designed to apply external electro-therapeutic stimulation to the genioglossus muscle in response to a predicted apnea event, was demonstrated by statistically significant improvements in several key OSA parameters (Table 3; Figs. 2 and 3).

**Table 3:** Clinical efficacy of Kalinix^®^ on key OSA parameters in study patients (*n* = 20). The table summarizes the effects of the external electro-therapeutic stimulation to the genioglossus muscle in response to predicted apnea events delivered by Kalinix^®^ on key OSA metrics. Treatment with Kalinix^®^ produced statistically significant improvements in the majority of the OSA parameters commonly used for evaluating the OSA severity and the treatment response

	Pre- Kalinix^®^, (Mean [SD])	Post-Kalinix^®^, (Mean [SD])	Absolute difference, (Mean change)	Change from baseline, (%)	Improvement from baseline, (%)	*p*-value
AHI	42.9 (24.0)	20,7 (16.8)	22.2	51.7	52.7	< 0.001
Duration apnea events (s)	20.6 (6.1)	19.8 (5.7)	0.8	4.0	15.2	≥ 0.05
Duration hypopnea events (s)	26.1 (4.3)	22.1 (4.0)	4.0	15.2	16.6	< 0.001
Total apnea events per hour	25.3 (22.8)	10.3 (12.9)	15.0	59.2	60	< 0.05
Total hypopnea events per hour	17.7 (8.3)	10.4 (7.4)	7.2	40.9	53	< 0.01
CT90	21.1 (27.1)	18.0 (27.0)	3.1	14.6	61.5	≥ 0.05
ODI	38.0 (21.6)	23.6 (18.6)	14.4	37.9	45.1	< 0.001
Heart rate	71.7 (4.9)	66.9 (10.3)	4.8	6.69	14	< 0.05
Respiratory rate	13.4 (0.6)	15.0 (2.2)	1.7	−12.8	-	< 0.01

The p-values were calculated based on the absolute values on baseline (pre-Kalinix®) vs. post-Kalinix® treatment and using paired non-parametric Wilcoxon signed-rank test. AHI apnea-hypopnea index, CT90 percentage of time with an oxygen saturation<90%, ODI oxygen desaturation index, SD standard deviation, SpO2% oxygen saturation

AHI is the most widely used metric in the classification of OSA severity and in the assessment of treatment response. Kalinix^®^ treatment promoted statistically significant improvement in mean AHI from baseline (42.9 vs. 20.7; *p* < 0.001), with improvements exceeding 50% from baseline (Table 1). In the study population, all patients achieved an improvement of ≥ 10% in AHI and, among them, 55% experienced improvements > 50%, which is the most commonly used threshold to classify a favorable OSA-treatment response.

In patients stratified by disease severity according to residual AHI at baseline, the response to Kalinix^®^ treatment was correlated with disease severity (*p* = 0.005; Fig. 2a). Similarly, the percentage of Kalinix^®^ responders (residual AHI < 15) was higher among patients with moderate OSA (100% of patients), with the proportion decreasing as disease severity increased [42.9% of responders with severe OSA (basal AHI 30–49) vs. 16.7% of responders with very severe OSA (basal AHI ≥ 50); Fig. 2B-D].

Kalinix^®^-treated patients also experienced a statistically significant improvement in other OSA-related parameters, including the duration of hypopnea events (*p* < 0.001), as well as the total events per hour (apnea *p* < 0.05; hypopnea *p* < 0.01) and the ODI (*p* < 0.001) (Table 3). Additionally, improvements in the variables evaluated that exceed 10% were observed in a substantial proportion of patients, including in 65% and 90% for the duration of apnea and hypopnea events, respectively, 90% for ODI, and 65% for CT90. Favorably, the heart rate also improved by 14% after Kalinix^®^ treatment (Table 3).

The investigator-defined clinical criteria were used to underscore population distribution based on treatment response (Fig. 3). According to the defined criteria, most of the population were classified as good treatment responders (45% of patients according to AHI and ODI criteria, 40% according to AHI and CT90 criteria and 45% according to AHI, ODI and CT90 criteria) (Fig. 3).

Together, all the above-mentioned results highlight the clinical relevance of Kalinix^®^ treatment, supporting its efficacy in the improvement of OSA severity and respiratory parameters.

### Patient satisfaction with Kalinix^®^ treatment

At the beginning of the study, 60% of patients rated their OSA as “bad” or “very bad”, which emphasizes the patient-experienced severity of condition. By the end of the study, 100% of patients were high or highly satisfied with Kalinix^®^ characteristics (ergonomics, portability) and 90% with performance (improvement in patient perceived-quality nocturnal rest). Additionally, 80% of the patients were highly satisfied with the overall treatment and, on a recommendation scale from 0 to 5, patients gave Kalinix^®^ a score of 4.8.

### Device tolerability and safety

The stimulation intensity required to initiate upper airway opening was within moderate ranges (mean ~ 42%) and moderate variability (a detailed description of electrostimulation intensity parameters and controlled safety ranges can be found in Supplementary Material, section of Technological device properties). The intensity associated with a maximal airway opening was higher, reaching 70% which indicate a gradual and controllable dose–response relationship. The mean patient-perception intensity was around 70%, which remarkably exceeds the values required to initiate airway opening. Additionally, the maximum tolerated device intensity was close to 95%, with low dispersion, being most patients’ records clustered above 90%, which reflects the high device acceptability. No adverse events or treatment-related side effects were reported during the study period.

Collectively, these findings suggested that physiological effect threshold of Kalinix^®^ occurs below conscious patient perception and supports a favorable safety profile.

### Comparative efficacy of Kalinix^®^ across other OSA treatment strategies

In addition to the patient-experienced benefits of Kalinix^®^, its clinical relevance in reducing AHI in comparison to other OSA-therapeutic strategies was also explored (Tables 4 and 5). Despite the well-stablished patient compliance issues, the proven efficacy of CPAP makes it the most commonly used treatment in OSA management [19]. A comparative efficacy analysis of Kalinix^®^ versus CPAP/APAP demonstrated that Kalinix is effective, providing a non-invasive and user-friendly solution that may promote treatment adherence (Table 4).

**Table 4:** Comparative efficacy of Kalinix^®^ vs. CPAP/APAP treatment strategies. The clinical efficacy of Kalinix^®^ in the reduction of AHI was evaluated in comparison with CPAP/APAP therapies, the most widely treatment used for OSA, based on data from previously published studies. Kalinix^®^ demonstrated clinically relevant reductions in AHI, supporting its potential as an effective and non-invasive therapeutic alternative

Study	Treatment under study	Basal AHI	AHI post-treatment
Meurice et al. (1996) [20]	CPAP	AHI, mean (SD) 40.5 (17.7) (auto-CPAP)	AHI mean (SD) 1.7 (1.2)
		AHI, mean (SD) 46.8 (22.3) (constant CPAP)	AHI, mean (SD) 2.6 (3.0)
Boyd et al. (2013) [21]	CPAP	AHI, mean (SD) 56.3 (22.6)	AHI, mean (SD) 4.3 (5.9)
Leng et al. (2021) [22]	CPAP	AHI, median (IQR) 28.8 (21.2–54.0)	AHI, median (IQR) 5.0 (4.2–6.0.2.0)
	APAP	AHI, median (IQR) 28.8 (21.2–54.0)	AHI, median (IQR) 5.5 (4.2–6.3)
-	Kalinix^®^	AHI 15–30, mean (SD) 23.56 (4.76)	AHI 15–30, mean (SD) 8.39 (3.53)
		AHI 15–50, mean (SD) 31.26 (10.51)	AHI 15–50, mean (SD) 14.52 (10.81)

*AHI* apnea-hypopnea index, *APAP* auto-adjusting positive airway pressure, *CPAP* continuous positive airway pressure, *IQR* interquartile range, *SD* standard deviation

**Table 5:** Indirect comparison of Kalinix^®^ efficacy based on treatment outcome parameters defined from studies on non-CPAP OSA treatments. The clinical efficacy of Kalinix^®^ with other non-CPAP OSA treatment strategies, including MAD, UPPP, and GA + HM was evaluated using treatment outcome parameters reported in previously published studies. These non-CPAP alternatives are currently associated with several limitations, such as invasiveness, postoperative morbidity or prolonged recovery times. In contrast, Kalinix^®^ represents a non-invasive therapeutic alternative that showed improved clinically relevant efficacy outcomes relative to established non-CPAP therapies

Study	Treatment under study	Treatment efficacy criteria	Treatment efficacy, %	Treatment efficacy post- Kalinix, %
Lee et al. (2013) [23]	MAD (mono-block)	AHI reductions >50%	77.4%	62.5%
	MAD (bi-block)		58.3%	66.7%
Tanyeri et al. (2012) [24]	UPPP	AHI reductions > 50%	46.9%	55.5%
Lee et al. (2011) [25]	UPPP	AHI reductions ≥ 50% Residual AHI ≤ 20	34.6%^*^	50.0%^β^
			31.8%^#^	52.6%^α^
Friedman et al. (2005) [26]	UPPP	AHI reductions ≥ 50% Residual AHI < 20	42.5% (moderate OSA) 26.5% (severe OSA)	100% 25.0%
Foltán et al. (2007) [27]	GA + HM	AHI reductions ≥ 50% Residual AHI < 20	74%	60.0%

*Patients with positional dependency; #Patients without positional dependency. Since positional dependency was not evaluated in the Kalinix® cohort, βpatients with an AHI>5 and αAHI>15 were selected for comparison with patients with and without positional dependency, respectively

AHI apnea-hypopnea index, GA genioglossus advancement, HM hyoid myotomy, MAD mandibular advancement device, OSA obstructive sleep apnea, UPPP uvulopalatopharyngoplasty

Additionally, in comparison with non-CPAP OSA treatments, Kalinix^®^ showed improved efficacy than other established OSA treatments, including MAD, uvulopalatopharyngoplasty (UPPP), and genioglossus advancement plus hyoid myotomy (GA + HM; Table 5).

Collectively, these results support the role of Kalinix^®^ as an effective, noninvasive and comfortable treatment option for OSA management for patients with compliance problems related to conventional treatments, such as CPAP and an alternative for patients with no compliance issues.

## Discussion

Globally, almost 1 billion people were affected by OSA, with prevalence exceeding 50% in some countries [28]. Although the consequences of untreated OSA and the impact on patient lives are well-documented, OSA remains significantly undertreated [6, 29]. The low adherence highlights the need for effective, safe, comfortable, non-invasive, and patient-friendly treatment strategies.

OSA is a multifactorial disease primarily driven by vulnerable pharyngeal anatomy and the reduced tone of the upper airway dilator muscles (e.g. genioglossus muscle), making the dilation of upper airways a promising therapeutic target for new therapeutic strategies [30]. Kalinix^®^ is an innovative, intelligent, safe, non-invasive, multi-sensor and self-adaptive medical device for the treatment of OSA developed to overcome the current limitations of OSA treatments by promoting upper airway opening through targeted electro-therapeutic stimulation of the genioglossus muscle.

CPAP remains the first-line treatment for patients with moderate to severe disease, however the high rates of treatment rejection and long-term compliance have driven the search for alternative treatments [6, 11–13]. Kalinix^®^ emerges as a non-invasive therapy with clinically proven efficacy in reducing disease severity and improving respiratory function. In fact, more than 50% of patients experienced improvements > 50% in AHI, with a satisfactory proportion classified as good responders.

Short-term improvements in objective respiratory parameters are considered a relevant indicator of treatment responsiveness in OSA. Consistent with this, our findings support the potential clinical relevance and Kalinix^®^ effectiveness under real-world conditions, although future larger, long-term and controlled studies will be valuable to further reinforce its therapeutic benefits. Notably, the study was designed to reflect real-world clinical practice, focusing on the device’s direct physiological effects under routine conditions rather than comparative efficacy (e.g., sham-controlled or CPAP group), consistent with its role as a real-world interventional evaluation. However, although the use of objective endpoints such as AHI during sleep reduces the influence of patient-related subjectivity, the single-arm design of this study does not allow full exclusion of confounding factors, including placebo effects and regression to the mean.

Despite the several technological innovations, over 30% of patients discontinued CPAP treatment after 5 years [6]. Kalinix^®^ was designed to meet patient needs, with 100% of patients satisfied with device ergonomics and portability, in addition to the improvements in patient-experienced perceived-quality of nocturnal rest. Another limitation of the CPAP is the need for individualized pressure titration to guarantee optimal pressure settings [31]. Contrarily, Kalinix^®^ offers a convenient, practical and effective solution with distinctive characteristics due to its multi-sensor and self-adaptive properties that adjust the electrostimulation to be applied in the genioglossus muscle in intensity, frequency and amplitude according to the needs of each apnea/hypopnea episode, guaranteeing an effective airway opening.

Currently, when conventional medical treatments fail due to inefficacy or low adherence, alternative treatments should be considered [6, 19, 32]. However, current alternatives, such as surgeries, MAD or hypoglossal nerve stimulation have limitations, such as high morbidity, long recovery times, low patient acceptance or limited efficacy to specific subgroups (e.g. BMI < 30 kg/m^2^, basal AHI level) [11, 19, 27, 33]. In contrast, our exploratory indirect comparison based on previously published literature suggests that Kalinix^®^ may achieve reductions in AHI within or above the range reported for non-CPAP OSA treatments. However, these observations should be interpreted with caution, as they are based on data from different studies with heterogeneous patient populations, study designs, and outcome assessment methods and they do not constitute direct evidence of comparative efficacy. Additionally, a satisfactory proportion of patients stratified by different disease severity (AHI ≤ 29 or 30–49) achieved residual AHI < 15 after Kalinix^®^ treatment, which aligns with the threshold used to guide patient indication for CPAP therapy and indicates that respiratory patterns improved during patient-sleep [34]. In contrast to other CPAP-alternatives, Kalinix^®^ provides a non-invasive, self-adaptive solution, which may be more accessible and acceptable to a broader population of patients with OSA.

The recognized necessity for new and emerging treatments drives us to develop an innovative approach based on the electrical stimulation of the upper airway dilator muscles. Since the ability of these muscles to respond to a respiratory challenge and maintain airway patency is pointed out as a key contributor of OSA, functional approaches focused at promoting genioglossus contraction have been shown to improve pharyngeal stability [7, 35, 36]. Among the current available strategies, other non-invasive technologies have shown substantial variability in neurostimulation approaches, using diverse stimulation parameters heterogenous implementations, which represents barriers to its successfully and consistent clinical applicability across OSA patients with different clinical profiles and varying severity of the episodes. Moreover, invasive strategies as the hypoglossal nerve electrical stimulation indirectly stimulates the genioglossus muscle and has exhibited a favorable efficacy profile in improving OSA severity [11, 37, 38]. However, the invasive nature of implantable devices that requires surgeries and postoperative recovery limits its clinical applicability and patient preference [11, 37, 38]. In contrast, Kalinix^®^ offers targeted, non-invasive and dynamic stimulation of the genioglossus muscle (according to the severity of each episode) which applies a real-time adjustment of stimulation intensity and timing, triggering an effective airway opening and a significant reduction in the number of apnea/hypopnea events. Due to its innovative, compact and patient-friendly design that allows patients to move freely during sleep without the inconveniences/discomfort of other OSA treatment systems, Kalinix^®^ offers a comfortable, efficacious and safe OSA treatment option that may contribute to achieving good long-term compliance and clinical outcomes.

### Study limitations and future directions

Kalinix^®^ has emerged as an innovative and recent therapeutic option for the treatment of OSA. However, given its novelty, there is a lack of data regarding its medium- and long-term use. Additionally, although the sample size and the demographic baseline characteristics of the cohort is not sufficient to evaluate its efficacy across a broader OSA population and specific population subgroups, the study demonstrates overall effectiveness of Kalinix^®^ in a cohort representative of a substantial proportion of OSA-patients treated in routine clinical practice. In the future, clinical trials with larger sample sizes, multicenter cohorts with more heterogeneous demographic and clinical profiles and controlled groups (e.g. sham-controlled and comparative trials) will be essential for subgroup analysis. Also, studies with extended follow-up periods of 3–6 months and longer, as well as close follow-up of the first patients using Kalinix^®^, are necessary to confirm the persistence of clinical benefits and to assess sustained patient adherence.

## Conclusion

OSA is a chronic disease that requires long-term multidisciplinary management, being pivotal that patients tolerate and adhere to long-term treatment [6]. More than 90% of OSA-treated patients expressed interest in exploring new treatments, suggesting that the current options do not fully satisfy patients’ expectations or needs [39].

Kalinix^®^ emerges as a promising, safe and patient-friendly treatment option for OSA patients that combine an effective and real time prediction of apnea/hypopnea events with a targeted genioglossus stimulation to promote airway opening. The device incorporates a multi-sensor system that monitors continuously relevant physiological signals, ensuring that stimulation is delivered effectively across different sleep stages and degrees of upper airway collapse. The significant improvements in key clinical parameters and the high rate of responders (≥ 50% AHI reduction) supports Kalinix^®^ efficacy. Besides, its non-invasive nature and its portable and comfortable design may contribute to improving long-term treatment compliance, especially for those who have difficulty in tolerating or adhering to other treatments, such as CPAP, or who need an effective alternative without the discomfort associated with traditional devices. Although these findings should be interpreted with caution due to the inherent study limitations, together the results provide encouraging evidence and support their further evaluation in larger controlled studies with extended follow-up periods.

## Supplementary Information

Below is the link to the electronic supplementary material.

Supplementary Material 1 (DOCX 27.0 KB)

## Data Availability

The data that support the findings of this study are available from Torytrans S.L., but restrictions apply to the availability of these data, which were used under license for the current study, and so are not publicly available. However, data is available from the authors upon reasonable request and with permission of Torytrans S.L.
