# Supplementary material for: Kalinix®, an innovative, intelligent, non-invasive, and self-adaptive medical device for effective OSA management
Source: Sleep Breath. 2026 Apr 29;30(2):148. doi: 10.1007/s11325-026-03687-y (PMC13128726; doi:10.1007/s11325-026-03687-y)
Supplement: Supplementary file 1 — Supplementary Material 1 (DOCX 27.0 KB) [file 11325_2026_3687_MOESM1_ESM.docx]

*Supplementary material*

**Descriptive characteristics and properties of Kalinix^®^ device**

**Overall description of the device**

Kalinix^®^ is an innovative, intelligent, non-invasive, multi-sensor and self-adaptive medical device designed for the real-time prediction of respiratory events and for the application of external electro-therapeutic stimulation to the lingual and perilingual muscles (genioglossus muscle) to avoid the episode whenever an apnea/hypopnea episode is predicted during sleep. This targeted stimulation favors the upper airway opening, improving airflow without causing perceptible discomfort to the patient.

**Clinical rationale for device design**

One key mechanism in obstructive sleep apnea (OSA) pathogenesis and responsible for recurrent apnea/hypopnea events during sleep associates the reduction in upper airway patency coupled with the reduced ability of the pharyngeal muscles, as the genioglossus muscle, to contract sufficiently to maintain upper airway opening during sleep [1, 2]. Among all pharyngeal airway muscles, genioglossus is by far the most extensively studied in OSA [2]. In healthy individuals pharyngeal muscle activation in response to increasing negative pressure swings and CO₂ accumulation restores airway patency during sleep [2]. However, this compensatory response is impaired in OSA patients, leading to airway collapse and further apnea/hypopnea events [2].

Based on the underlying-OSA pathophysiological characteristics, Kalinix^®^ was designed to predict apnea/hypopnea events (airflow limitation) mediated by excessive upper airway muscle relaxation. Upon events prediction, Kalinix^®^ applies electrostimulation to genioglossus muscle, increasing the upper airway caliber and improving airflow.

**Technological device properties**

Kalinix^®^ is composed of central device, pressure sensor, thermistor and electrodes. The electrodes are placed on the patient’s skin and connected to a phone-sized device (92 x 85 x 25 mm, ~120 g) with battery (rechargeable: 7.4V DC, duration: 8-22 h) that is attached to the arm or chest with an easily adjustable strap. Kalinix^®^ was designed to allow patients to move freely during sleep and avoid the inconveniences/discomfort of other OSA treatment systems. The central device contains a processor responsible for predicting the apnea/hypopnea events and the electronics required to generate a real-time electrostimulation signal to prevent the events.

The apnea/hypopnea events are predicted based on the sensorial information collected from the patient (respiratory rate and respiratory air temperature, which are continuously acquired via a nasal cannula) and analyzed in real time by AI algorithm, which identifies respiratory patterns to predict apnea/hypopnea episodes.

Upon event prediction, Kalinix^®^ applies electro-therapeutic stimulation (waveform: rectangular, bipolar, symmetrical, out of phase; maximum output current of the electrode: <20mA [100 kΩ load resistance]) to the genioglossus muscle, leading to upper airway opening to provide sufficient airflow and prevent upper airway occlusion. The electrostimulation signal (signal frequency: 23 Hz; pulse duration: 200 µs, Output Voltage: 77.6 V [square pulse] and 65.6 V [pseudo triangular pulse]) provided by Kalinix^®^ varies in intensity, frequency and amplitude according to the needs of each episode, with predefined and controlled safety ranges (current intensity: 0–20 mA; voltage amplitude: 0–77.6 V; stimulation frequency: event-dependent and dynamically modulated according to the occurrence and characteristics of apnea/hypopnea episodes). Within these ranges, the stimulation signal is not applied at a fixed level; instead, the intensity and amplitude delivered by Kalinix^®^ increase progressively during each respiratory event until resolution is achieved or until individual safety and tolerance thresholds are reached. More severe or deeper events are associated with stronger and longer-lasting stimulation, while remaining within the established limits. As an additional safety measure, the electrostimulation will never last more than 8 seconds.

The stimulation ranges were established through a stepwise clinical development process, integrating prior safety evidence with clinical optimization in the present trial. In this study, stimulation parameters were further refined during a pre-treatment, in-clinic titration phase, ensuring both physiological efficacy and patient tolerability.

*Patient-specific calibration*

Patient-specific safety and tolerance thresholds were defined during individualized titration prior to home use. For each patient, perception threshold and tolerance threshold were identified, and the stimulation intensity was selected within the individual-specific window defined by these thresholds. Simultaneously, upper airway opening is evaluated in real time using 3D/4D ultrasonography and elastography, allowing objective confirmation of genioglossus activation and airway patency. Individual optimal stimulation parameters are defined during the initial clinical visit (day 1) and confirmed on day 2 to verify persistence of the physiological response, before initiating the home treatment phase. During sleep, the system continuously monitors respiratory signals and for each detected or predicted episode, Kalinix® generates a stimulation waveform adapted to the severity and depth of the event, progressively increasing intensity and amplitude until the episode resolves, within the predefined individual safety limits. The algorithm dynamically adjusts stimulation timing, intensity, and duration according to the recorded breathing parameters, allowing effective adaptation to changing physiological conditions throughout sleep.

*AI algorithm*

The AI algorithm predicts in real time respiratory patterns based on a historical record of successful stimulation events, analyzed using a sliding time-window approach. Incoming respiratory signals are continuelsly compared with previous effective patterns.

The algorithm dynamically prioritize combinations of stimulation parameters (such as intensity, frequency, or pulse duration) that have shown positive outcomes under similar physiological and respiratory conditions within the most recent historical window, rather than rely on fixed rules or prior assumptions about individual patient response.

This adaptive strategy enables personalized real-time adjustment while maintaining patient safety and minimizing unnecessary stimulation. A detailed mathematical or architectural description of the algorithm is beyond the scope of this manuscript and includes proprietary elements; however, the principles described reflect the conceptual framework governing event prediction and stimulation optimization.

*User interface and data tracking*

Kalinix^®^ is equipped with a screen that displays relevant data, such as real-time AHI and device operating time, as well as memory card to record nighttime data. Additionally, Kalinix^®^ is accoupled to a digital platform that displays the results collected on the memory card and allows other relevant patient information, such as tracking, disease condition, treatment progress, and more. Together, Kalinix^®^ provides a comprehensive patient data management tool that improves OSA monitoring and supports personalized treatment adjustments in a comfortable and non-invasive manner.

**Device viability and safety**

Kalinix^®^ safety and device viability were first evaluated in a pilot study (Ethics Committee: 00718_TOR_PROT_EC) to explore device ability to be used effectively and safely in real-world clinical settings. Preliminary data indicate that Kalinix^®^ electrostimulation did not affect heart rate, nor did it cause an increase in skin temperature or skin changes after electrode removal. At the highest sensitivity threshold, subjects reported only tingling sensation (intensity 0-2), and only 50% of the subjects reported pain at the tolerance threshold (intensity 7-7.5). Additionally, in 70% of subjects, a visible thickening of the tongue was detected around the tolerance threshold, although it was not perceived by the subjects. No other adverse effects were observed.

Kalinix^®^ viability was conducted in two phases. In the first phase, 3D/4D sonography (in sitting and supine positions; MyLab™X90, Esaote's ultrasound) and PSG (oropharyngeal region, NOX T3) were used to explore the morphological and functional impact of Kalinix^®^ electrostimulation on upper airway opening in OSA patients. This phase was also vital for the determination of the most suitable locations for electrode positioning. It was observed that Kalinix^®^ electrostimulation of tongue muscles, predominantly the genioglossus muscle, induces painless contraction and contractile retraction, allowing a temporary separation between the base of the tongue and the surrounding structures, thereby increasing the caliber of the upper airway in 100% of cases. During the procedure electrostimulation was tolerated in all patients.

In the second phase, a comparative analysis was conducted to evaluate the ability of Kalinix^®^ to detect breathing patterns *vs.* conventional polysomnography (PSG; NOX T3). Apnea-hypopnea index (AHI) measurements achieved with Kalinix^®^ showed a strong and statistically significant linear correlation with the conventional PSG, with coefficient of 0.97 (p<0.025).

These findings confirm that Kalinix^®^ is ​​an accurate, reliable and equally valid tool for predicting apnea/hypopnea events and evaluate AHI levels, with a prediction range with 80-92% of precision (risk of error of 2.5%).

**References**

1. Eckert DJ, Malhotra A (2008) Pathophysiology of adult obstructive sleep apnea. Proc Am Thorac Soc 5:144–153. https://doi.org/10.1513/pats.200707-114MG

2. Edwards B, White D (2011) Control of the Pharyngeal Musculature During Wakefulness and Sleep: Implications in Normal Controls and Sleep Apnea. Head Neck Suppl 1:S37-45. https://doi.org/10.1002/hed.21841.Control
